# ASSIST in Pitjantjatjara: Protocol for a randomised crossover validation study among Aboriginal and Torres Strait Islander Australians

**DOI:** 10.1016/j.conctc.2025.101532

**Published:** 2025-08-07

**Authors:** Matthew W.R. Stevens, Sue Bertossa, Dominic Barry, Chris Holmwood, KS Kylie Lee, John Marsden, Matt Pedler, Mark Thompson, Scott Wilson, Robert L. Ali

**Affiliations:** a School of Biomedicine, The University of Adelaide, Adelaide, SA, Australia; b Flinders Wellbeing Centre, Salisbury, South Australia, Australia; c School of Psychology, University of South Australia, Adelaide, SA, Australia; d Centre for Alcohol Policy Research, La Trobe University, Melbourne, Victoria, Australia; e National Drug Research Institute, Curtin University, Perth, Western Australia, Australia; f Faculty of Medicine and Health, The University of Sydney, Camperdown, New South Wales, Australia; g Burnet Institute, Melbourne, Australia; h Edith Collins Centre, Translational Research in Alcohol Drugs and Toxicology, Drug Health Services, Sydney Local Health District, Australia; i NIHR Maudsley Biomedical Research Centre (BRC), Institute of Psychiatry, Psychology and Neuroscience, King's College London, London, United Kingdom; j Drug and Alcohol Services South Australia (DASSA), Magill, SA, Australia; k Aboriginal Drug and Alcohol Council South Australia Aboriginal Corporation, Underdale, SA, Australia

**Keywords:** SBIRT, Substance use disorders, Dependence, Aboriginal health and Wellbeing, Alcohol, Tobacco and illicit drug use, Prevention, Harm minimisation, Pitjantjatjara, ASSIST

## Abstract

**Background:**

Substance use significantly contributes to disease burden among Australians, with harms exacerbated among Aboriginal and Torres Strait Islander peoples by colonisation-related factors like stigma and trauma. Addressing this gap requires culturally acceptable, valid and reliable screening tools, available in a familiar language to the participant, to identify and provide support for those at-risk. This protocol describes a study aimed at validating a culturally-adapted screening tool — the Alcohol, Smoking and Substance Involvement Screening Test (ASSIST) — into Pitjantjatjara, to detect risk of substance-related harm.

**Methods:**

Recruitment will occur at a variety of Aboriginal health and welfare settings across remote, rural and urban South Australia. Eligible participants (aged 18–65) will be briefed and, upon consent, randomly complete the ASSIST app on an iPad and a semi-structured, yarning-style diagnostic interview (see endnote 1) with a health professional and Pitjantjatjara interpreter. The interview will assess for a range of clinically-defined substance use disorders (based on DSM-5-TR and ICD-11 criteria). All participants will be asked to complete the app a second time (between 7 and 28 days) to assess reliability, while a subset of participants at highest-risk will also undergo specialist evaluation from an independent clinician, as a second check for validity.

**Discussion:**

Valid and reliable assessment tools are essential for detecting risky and harmful substance use. If valid, this app has the potential to contribute to community-led efforts to bridge the health gap by addressing modifiable health risk factors.

**Trial registration:**

ANZCTR: ACTRN12625000413426. Open Science Framework pre-registration: https://doi.org/10.17605/OSF.IO/GNZAY.

**Version control number:**

Protocol version 1.1, June 23, 2025.

## 1 Introduction

Alcohol, tobacco and illicit drug use remains an ongoing public health concern in Australia. These substances feature in the top-10 risk factors for preventable disease burden [1]. For Aboriginal and Torres Strait Islander peoples (respectfully termed “Indigenous Australians” herein), mental health and substance use disorders account for nearly 25 % of this burden [2]. Regrettably, a health gap persists between Indigenous and non-Indigenous Australians, with individual ‘health risk factors’ (which includes substance use) accounting for nearly 20 % of this gap [3,4]. Substance use behaviours are modifiable with appropriate support. In the Indigenous Australian context, this requires community-led approaches that nurture cultural identity, cultural participation, and ongoing connection to land, country, community, and language [5].

While many Indigenous Australians maintain good health throughout much of their lives, their average health-adjusted life expectancy is about 8 years shorter than non-Indigenous counterparts [5]. Indigenous Australian communities have been working to reduce the health gap over many years [6]. However, unique inequities experienced by Indigenous Australians, dating back to British colonisation in the 18th century, include stigma, discrimination, racism, and intergenerational trauma. These factors continue to impact on health and livelihood [[7], [8], [9], [10], [11]], and compound susceptibility to harm from problematic substance use. Evidence on alcohol consumption among Indigenous Australians is mixed. Some studies suggest Indigenous Australians may be less likely to consume alcohol compared with non-Indigenous Australians [12,13]. However, recent representative community sample studies have shown rates of alcohol consumption in-line with patterns of consumption among the general population in Australia [14]. Other evidence indicates higher rates of risky consumption among those who do consume alcohol [15]. Additionally, nearly one-in-three (29 %) Indigenous Australians report smoking daily; while more than 25 % report using illicit drugs in the past 12-months, predominantly cannabis [16]. Reducing overall risky and harmful substance use requires a tailored public health approach to address health inequities.

Identifying at-risk individuals early allows tailored interventions to reduce substance use escalation and related harms [17]. However, effective secondary prevention strategies, such as screening brief intervention and referral to treatment (SBIRT), rely on accurate and culturally sensitive assessment tools [18,19]. Many existing tools lack sensitivity for use in Indigenous Australian contexts. This is primarily due to language barriers, literacy issues, cultural nuances, and differing worldviews [20,21]. Until recently, few tools were available in language to detect risky substance use, beyond alcohol and tobacco [13,22]. In 2021 some of the authors of this protocol were involved in a community-led project to translate and culturally adapt one such instrument, the Alcohol, Smoking and Substance Involvement Screening Test (ASSIST) [23] into Pitjantjatjara for use in Indigenous Australian communities [24]. The ASSIST was selected for its intent as a secondary preventive approach that screens for all substances and connects that risk assessment to a purpose-built brief intervention.

Recognising the need for cultural adaptation beyond a simple translation, the Pitjantjatjara ASSIST has undergone modifications to enhance its appropriateness for use in Indigenous Australian settings. These include refining language, modifying the structure of some individual the items, and adding audio components in Pitjantjatjara or English to aide comprehension, along with visual elements, including graphics designed by an Aboriginal Australian artist. Given these modifications, validating the ASSIST's reliability and accuracy for detecting risky and harmful substance use is essential. This protocol outlines an order-randomised crossover validation study aimed at assessing the diagnostic accuracy, psychometric performance, and cultural acceptability of the newly developed digital instrument among a sample of Pitjantjatjara-speaking Indigenous Australians, using a gold-standard clinical interview.

## 2 Methods

### 2.1 Indigenous Australian leadership

The entire project, including the translation and cultural adaptation of the instrument previously reported on [24], was co-designed with Indigenous Australian health professionals (MT, SW), Indigenous Australian researchers (DB) and non-Indigenous Australian researchers (RA, MS, SB, CH, KL, JM, MP). The tablet-based app will be implemented by both Indigenous and non-Indigenous Australian stakeholders. The culturally adapted intervention (currently under development) will be co-designed with stakeholders including Indigenous Australian health professionals, addiction medicine specialists, community leaders, and researchers (Indigenous and non-Indigenous Australian).

### 2.2 Participants and study design

The target population for this study are Pitjantjatjara-speaking adult men and women who use alcohol, tobacco or other drugs. We will recruit participants with the full spectrum of substance use behaviours, including those who are abstaining, those who are using recreationally, through to those who are clinically dependent. The purpose of doing so is to identify whether the instrument is capable of differentiating between risk severity among the target group.

#### 2.2.1 Inclusion and exclusion criteria

Eligible participants must self-identify as Pitjantjatjara- or English-speaking Anangu (meaning ‘people’ in Pitjantjatjara-Yankunytjatjara, the Traditional Owners of the APY-lands across South Australia, Northern Territory, and Western Australia) and be aged 18–65. Since participants are also required to conduct a diagnostic interview (which will be conducted in English with the aid of a translator), fluency in either (spoken or written) English or Pitjantjatjara is required. Participants who appear intoxicated at the time of the interview will not be eligible to participate in the study, but will still be able to try out app should they choose. All participants will be reimbursed for their time with store vouchers for local grocery stores (e.g., Coles, Woolworths or Foodland/IGA; depending on site) of up-to $90 total value ($30 for completing the ASSIST, $30 for the interview, and a final $30 for completing the follow-up). Grocery store vouchers were selected in this case because, by their nature, cannot be redeemed for alcohol or tobacco or other substances, aligning with ethical considerations for substance use research, and community preferences identified during community consultation.

#### 2.2.2 Recruitment

A stratified sampling strategy will be employed to target individuals across the spectrum of risk for each of the five substances included in the Pitjantjatjara ASSIST (“*the app*”, i.e., alcohol, tobacco, cannabis, methamphetamine and inhalants). Since *the app* focuses on these five substances only, and uses existing WHO-ASSIST 3.1 cut-off scores to determine three corresponding levels of risk for each (i.e., low, moderate or high), there are a total of 15 (5x3) possible substance/risk combinations. We will aim to recruit a minimum of 40 individuals to satisfy each of those outcomes (e.g., 40 participants each for low, moderate and high-risk, for each of the substances This could require up to 600 participants, though the total will likely be lower since many use multiple substances. Recruitment will occur until we meet the minimum number for each group, or until the investigators deem it unnecessary to continue. Further details related to the power calculations is found in section 3.5. *Sample Size and Power Calculations* below.

To capture a broad range of participants, we will recruit from a variety of health and welfare settings. The goal is to recruit individuals who are likely to be engaged in low and/or moderate risk alcohol, tobacco or other drug use (e.g., from general health and welfare settings), and those who are more likely to be engaged in use which places them at higher-risk of dependence (e.g., from withdrawal services or other inpatient/outpatient treatment settings). Recruitment will occur at seven different sites across South Australia, by word-of-mouth at each service or setting, where Aboriginal health workers will approach potential participants directly.

#### 2.2.3 Sites

Several sites across South Australia are proposed for recruitment. These include the withdrawal service at Drug and Alcohol Services South Australia (DASSA), and the managed alcohol program at DASSA's Glenside Service; Flinders Wellbeing Centre (Salisbury, South Australia); and sites managed by The Aboriginal Drug and Alcohol Council (ADAC; Ceduna, South Australia). Participants may also be recruited from DASSA's Assertive Outreach Program that occurs at various sites within the APY-lands.

#### 2.2.4 Procedure

Eligible participants will be approached by an Aboriginal health and welfare worker (“health worker”) at one of the recruitment sites. In most cases, the health worker will be known to the participant, and so an independent research assistant (“independent researcher”) will be introduced to potential participants. For cultural reasons, the health worker and independent researcher will be the same gender as the participants. Prospective participants will be informed by the independent researcher of the study's aims, their right to choose whether or not to participate voluntarily; their right to withdraw at any time should they wish to proceed; and that any refusal will not impact on care they receive from staff at each service (consenting and non-consenting participants will receive the same care as usual from the services they attend). Participants may receive standard care from recruitment sites alongside study interventions, which may be stopped if they request withdrawal or show significant distress, as determined by the research team. No ancillary studies are planned; thus, no additional consent provisions are required.

The independent researcher will also be fluent in Pitjantjatjara, and so will also act as an interpreter/translator to provide additional information or context to participants. The iPad tablet contains both *the app* and the interview. Both components contain the embedded information sheet at the front-end, which participants must read entirely before being allowed to provide consent. Those who agree to proceed will then be randomly allocated to one of two groups, app or interview (using a random sequence generator built onto the iPad). The individual initiates the randomisation sequence, which conceals allocation sequencing until assignment.

Once informed consent has been given via the iPad, participants randomised to *the app*-first group will be given a guided visual and tactile demonstration of how to use *the app* by a member of the research team (either health worker or interpreter) and then be asked to complete the instrument on their own. The research team members will sit a short distance away in case there are questions or technical issues with *the app*. Participants will then be provided with a $30 voucher and given a short break before completing the interview.

Participants randomised to the interview-first condition will sit down with the independent researcher/interpreter to conduct the yarning-style interview. At the outset, participants will be asked the same four initial anonymous identifier questions for the purposes of matching data from the interview and app. All interviews will be conducted face-to-face. All data will be recoded in REDCap. Participants will receive $30 voucher for completing the interview. Participants will then be given a short break before completing *the app*.

Upon completion, a follow-up appointment will be scheduled with participants for within fourteen days. At follow-up participants will also be asked to complete *the app* assessment component again (i.e., but not the interview), in order to assess *the app*'s test-retest reliability. To maximise follow-up retention, participants will receive a final $30 voucher, but only upon completion of the final assessment.

For participants taking part in the DASSA withdrawal service (i.e., those representing a higher risk group), an independent clinical assessment, conducted by an Addiction Medicine Registrar employed at the service, will also be used as an additional check of validity. The independent assessment form will include the same unique participant code (see preceding *2.3.1. Unique participant code* section), as well as participants’ age and gender. The assessment will be recorded on a digital form in REDCap and will be completed by the doctor at the service.

#### 2.2.5 Adverse events

In the event that participants experience distress, a research team member will be available to provide immediate assistance and facilitate access to support services. Should participants express personal concerns or exhibit signs of distress, prompt support will be provided. If a participant wishes to withdraw from the study, we will help them contact their healthcare provider or access immediate care. Emergency services and mental health triage contact information are included in the Pitjantjatjara ASSIST app, which also offers a directory of support services, enabling participants to access relevant phone numbers or websites.

### 2.3 Data collection and instruments

#### 2.3.1 Unique participant code

Given there are multiple stages of this study (*the app* assessment, diagnostic interview (with independent evaluation for the high-risk group), and follow-up *app* assessment), it is necessary for the researchers to match participants responses through each stage. Since *the app* and diagnostic interview refer to sensitive information, it is also necessary to avoid capturing participant information that could be used to identify them. To mitigate the risk of sensitive information becoming identifiable, a unique participant code that anonymises the participant and their data to everyone, including the research team will be created automatically during both the app and interview stages.

The unique code will be generated automatically by the participant, based on their answers to the following four questions: what is your birth month? (coded 0–12); what is the first letter of your mother's first name? (coded A-Z); how many children do you have? (coded 0–99), and; what is the first letter of your first name? (coded A-Z). Participants will provide responses to these four questions anonymously within *the app*, or interview at the consenting stage, and the participant's unique combination of integers and digits will be concatenated to form each participant's unique code. So, for example, a person born in August (08), who's mother's name was Jenny (J), with three children (3), and who's own name was Mark(M), would be 08J3M. Structured in this way, anonymous data will be stored for each participant, preventing any risk of identification. Unique codes will be generated and stored automatically in the REDCap database.

#### 2.3.2 Pitjantjatjara ASSIST

The Pitjantjatjara ASSIST is a translated and culturally-adapted version of the ASSIST (ref for original ASSIST). Like the full ASSIST, the Pitjantjatjara ASSIST is an 8-item questionnaire designed to assess risk of harm from substance use disorders. However, unlike the full ASSIST, the adapted instrument only focuses on five primary drugs of concern in the Pitjantjatjara community, which include alcohol, tobacco, cannabis (“marijuana”), methamphetamine (“ice”), and inhalants. Additionally, unlike the full ASSIST, the adapted instrument steps *the app*-user through items 2–7 for each substance sequentially. The scoring algorithm for the adapted instrument remains unchanged. Upon completion of the instrument, individual's substance-specific scores for each inventory (SSI) will be summed, and presented with a corresponding calculation of risk. The English version of the ASSIST has been validated in a variety of health and welfare settings and populations [25]. A detailed description of the app, including design, flow, and features is available as an appendix (see supplementary materials).

#### 2.3.3 Diagnostic interview

To validate *the app* against a clinical gold-standard for diagnosing DSM-5 [26] and ICD-11 [27] substance-related disorders; a diagnostic interview will also be conducted with each participant, on the same day as they complete the ASSIST app. The interview is based on the DIS-SAM [28], which has been modified to identify the presence or absence of clinical symptoms for ICD-11 (for hazardous use, harmful use, and dependence) and DSM-5-TR substance use disorder, and includes assessments at both the past 12-month, and past 3-month intervals.

The interview will be conducted by either a male or female health professional (matched to participant's gender). The health professional will understand Pitjantjatjara culture and context but may not be Aboriginal or fluent in the language. Therefore, in addition, an Aboriginal man or woman will also be present at all times to aid interpretation of questions. The choice to include both Aboriginal and non-Indigenous Australian healthcare workers was taken to mitigate the risk that the differences in culture will impact the quality of the assessment [20,21,29]. Interviewers assessments will be based on use in the past 90, and 365 days.

The interview will follow a semi-structured *yarning*-style approach based on a clinical yarning framework. Clinical yarning is a culturally secure framework for clinician-client communication with Aboriginal and/or Torres Strait Islander peoples [30]. This approach has been developed specifically to address the well-documented communication barriers that exist between Aboriginal and Torres Strait Islander peoples and mainstream healthcare services, which often result in misdiagnosis, under-diagnosis, and poor therapeutic engagement [31]. To ensure comprehensive assessment of DSM-5-TR and ICD-11 substance use disorder criteria while maintaining cultural appropriateness, the interview contains a clinical checklist that maps yarning conversation topics to specific DSM-5-TR and ICD-11 criteria. Interviewers will be encouraged to use their discretion to introduce culturally appropriate metaphors, examples, or explanations to support participant understanding and comfort, while still ensuring that all required clinical content is covered. The clinical yarning framework has been shown to reduce communication barriers, and promote greater levels of trust and engagement, while maintaining diagnostic rigour [[31], [32], [33]]; and has been successfully implemented in similar contexts [34]. Where possible, interviews will be kept to 30 minutes or less to minimise participant burden.

#### 2.3.4 Independent clinical evaluation

Participants recruited from the DASSA withdrawal service will also undergo an independent clinical evaluation from an addiction medicine specialist, trained to diagnose the presence or absence of ICD-11 and DSM-5-TR disorders related to substance use. The registrar will be blinded from the outcome of the earlier assessment and will provide a clinical opinion as to whether the individual in their care meets the diagnostic threshold for hazardous use, harmful use or dependence (ICD-11) and substance use disorder (DSM-5-TR) for each of the five substances. In all cases, the individual will be known to the clinician (as a patient of the service).

### 2.4 Data and protocol monitoring

A data monitoring team — comprised of the first and last author — will work closely with investigators to monitor trial conduct and safety, assess risks and benefits, and make recommendations to protect the participants of clinical trials. To ensure adherence to study protocols, a small group of three-to-five research assistants will be trained extensively to conduct the assessments and interviews. A copy of the study protocol will be provided to each research assistant, interviewer, and interpreter. A regular monthly check in with the group is scheduled to discuss any issues with data collection and study protocol. Any protocol deviations will be reported in writing to each ethics committee within 72 h. No steering committee or other oversight groups are involved beyond the data monitoring team. Trial conduct will be audited monthly by the data monitoring team to ensure protocol adherence.

## 3 Statistical approach

A detailed statistical analysis plan is available as an appendix (see supplementary materials). Once all data has been collected, a cleaned, de-identified dataset will be stored within an online repository [35]. The primary outcomes from this study include *the app*'s reliability, validity, diagnostic accuracy, and cultural acceptability. Reliability will be assessed according to internal consistency and test-retest. Validity will be assessed according to both concurrent and discriminative validity against a gold-standard clinical interview outcomes and symptom counts. A small subset of participants will also receive an independent evaluation from a Specialist Addiction Medicine Registrar, which will form the basis of an additional check for concurrent validity. Diagnostic accuracy of the instrument will be assessed based on a range of indices and receiver operating characteristics (ROC) curve analysis. Finally, *the app* will also be assessed for its overall cultural acceptability. As part of initial data screening, count distributions, dispersion characteristics, and relevant modelling assumptions will be examined. *Note*: if any of the following assessments indicate substantial deviation from planned analytic assumptions (e.g. zero-excess, or non-linearity), appropriate modifications to the analysis strategy will be implemented and fully described in the final analysis report.

### 3.1 Reliability

#### 3.1.1 Internal consistency reliability

Internal consistency reliability will be assessed for each of the five substances using McDonald's Hierarchical Omega (ω_*h*_). Hierarchical Omega estimates the reliability of a general factor in potentially multidimensional scales [36,37] which is expected to be the case in this analysis. Additional coefficients (Total Omega [ω_*T*_] and Cronbach's Alpha [α]) will also be calculated and reported for completeness. Importantly, since SSIs contain a small number of items (five for tobacco, or six for the remaining substances), bootstrapping will be used to approximate the distribution of the all coefficients within a given range, using *n* = 10,000 samples to accommodate biases related to too few scale items [38]. This will calculate a distribution of values for each coefficient, which we could reasonably expect from our sample. A significant (two-tailed *p* < 0.05) ω_*h*_ coefficient ≥0.7 will indicate acceptable internal consistency reliability.

#### 3.1.2 Test-retest reliability

Test-retest reliability will be assessed by asking participants to complete the app twice within a period of between 7 and 28 days. Reliability will be indicated through Intraclass Correlation Coefficients (ICC_3,1_ [two-way, mixed-effects, absolute agreement, single rater]) at the scale level. ICC values for each item will also be calculated and reported for completeness. Significant (two-tailed *p* < 0.05) ICC ≥0.5 will indicate acceptable agreement [39,40].

### 3.2 Validity

#### 3.2.1 Concurrent validity

Concurrent validity will be assessed in two ways. First, a confirmatory approach will assess the partial-order (Spearman's Rho [ρ]) correlation between ASSIST SSI scores for each scale, and the total number of DSM-5-TR and ICD-11 diagnostic symptoms reported for that substance. The use of partial correlations will help to control for the effects of other substances and demographic factors. Benjamini-Hochberg (BH) correction will be used for each correlation to control the False Discovery Rate (FDR) [41]. BH-corrected significant (FDR <0.05) partial correlation ρ ≥ 0.5 will indicate concurrent validity.

The second (exploratory) approach will involve assessing the relationship between ASSIST SSI scores and the number of DSM-5-TR/ICD-11 symptoms through linear and quadratic regression models. For every substance, a regression plot will map the number of ICD-11/DSM-5-TR symptoms reported (vertically along the y-axis) as a function of the ASSIST SSI score (horizontally on the x-axis). As there are five substances, and two distinct classification systems, a total of 20 separate regression models will be carried out (10 linear, and 10 quadratic). For each substance, a significant linear and/or quadratic relationship (*p* < 0.05) is expected to be found between the number of symptoms reported and the average ASSIST scores. Model fit indices will be reported, and models will be compared using AIC and BIC values, to prevent overfitting. Models with the lowest AIC/BIC values will be selected.

A final check for concurrent validity (depending on sufficient data) will investigate the relationship between ASSIST SSI scores for each substance, and the outcomes from the independent clinical assessment by an Addiction Medicine Specialist. For each substance, a logistic regression model will be used to assess the probability of reaching a diagnosis, per unit score increase on the ASSIST. For each substance, a significant model (*p* < 0.05) is expected to be found between the ASSIST scores and the diagnosis probability.

#### 3.2.2 Discriminant validity

Discriminant validity assesses the extent to which an instrument can differentiate between two (or more) theoretically independent groups. In this study, groupings will be determined by ICD-11 and DSM-5-TR diagnostic categories respectively. Differences between scores as a function of diagnostic classification will be assessed using initial one-way ANOVA, with a series of planned pairwise t-tests to confirm group-level differences. Ten separate ANOVA models will assess whether differences exist in the distribution of ASSIST scores between groups for each substance and classification system (i.e., 5-substances x 2-systems). Table 1 below outlines the planned analysis for the two tests.

**Table 1:** One-way ANOVA models assessing group differences for ICD-11 (Model set 1) and DSM-5-TR (Model set 2).

Model set	Diagnostic Severity Group (per substance)			
1	No disorder	Hazardous use	Harmful use	Dependence
2	No SUD	Mild SUD	Moderate SUD	Severe SUD

In the first set of models, score distributions will be compared based on ICD-11 diagnostic classifications for each substance, while the second set of models will compare score distributions based on DSM-5-TR diagnostic classifications. If a significant omnibus test is found (i.e., *p* < 0.05) for each substance/classification, as expected, three planned (*a priori*) pairwise comparisons using BH-correction (setting FDR <0.05) will assess the magnitude and direction of those score differences.

Table 2 below outlines the planned comparisons to be carried out. For each substance, the first set of planned comparisons will assess the ability of the Pitjantjatjara ASSIST SSI to discriminate between the absence of DSM-5-TR or ICD-11 disorder, versus mild SUD and hazardous use, respectively. The second set of comparisons will assess the ability of the SSI to discriminate between mild vs moderate SUD; or between hazardous vs harmful use, respectively. The third set of comparisons will assess the ability of the SSI to assess risk between moderate vs severe SUD; and between harmful use vs dependence, respectively (see Table 2).

**Table 2:** Planned (A vs. B) pairwise comparisons of ASSIST scores by ICD-11/DSM-5-TR diagnoses.

Planned comparisons		No disorder/No SUD	Hazardous use/Mild SUD	Harmful use/Moderate SUD	Dependence/Severe SUD
1	Low risk	A	B	*excluded*	*excluded*
2	Mod. risk	*excluded*	A	B	*excluded*
3	High risk	*excluded*	*excluded*	A	B

### 3.3 Diagnostic accuracy

The second step of the analytic approach will be to update the score thresholds for determining risk. Receiver Operating Characteristics (ROC) curve analysis will be used to define the most appropriate cut-off scores for stratifying low, moderate, and high-risk use. Based on the new thresholds, outcomes from the diagnostic interview outcomes will be used to determine the number of true positive (TP), true negative (TN), false positive (FP), false negative (FN) cases for each diagnosis within the sample. Where trueness or falseness is determined by the interview, and positivity or negativity is determined by the ASSIST risk category, based on the theoretical alignment between low-risk ASSIST and mild SUD/hazardous use; moderate-risk ASSIST and moderate SUD/harmful use; and high-risk ASSIST and severe SUD/dependence. For example, TP in the context of ICD-11 dependence would represent the number of high-risk ASSIST cases (i.e., positivity) who were diagnosed with dependence (i.e., trueness) on the interview. Similarly, TN would represent the number of low and moderate risk ASSIST cases who were not diagnosed with dependence. TP, TN, FP and FN parameters will then be used to calculate sensitivity, specificity, positive predictive value (PPV) and negative predictive value (NPV) for each substance, at each risk threshold, i.e., for DSM-5-TR between no disorder vs. any disorder (low risk threshold); between no disorder/mild SUD vs. moderate/severe SUD (moderate risk threshold); and between non-severe vs. severe SUD (high risk threshold). Parallel analysis will occur based on ICD-11 classifications.

#### 3.3.1 Sensitivity and specificity

Sensitivity describes the proportion of correctly identified true cases, and thus can be calculated by dividing the number of true positive cases by the total number of positive cases in the sample (TP/TP + FN). On the other hand, Specificity describes the proportion of correctly identified negative cases, and thus can be calculated by dividing the number of true negative cases by the total number of negative cases in the sample (TN/TN + FP). ROC curves can be calculated for each substance and level of risk by plotting sensitivity on the x-axis, and (1-specificity) on the y-axis. The point at which the distance from the ROC curve (AUC) to the diagonal is maximised (Youden's *J*) can then be used to identify the most appropriate cut-off scores for each substance, at each level of risk. In effect, AUC describes the probability, if given one TP, that that TP will score higher than one TN on the test. The criterion for AUC will be set at ≥ 0.71, to indicate at least better than acceptable accuracy [42].

#### 3.3.2 PPV and NPV

PPV describes the probability that an individual will be a true positive case, given a positive screen, and thus represents the proportion of true positive cases out all those receiving a positive screen (TP/TP + FP). On the other hand, NPV describes the probability that an individual will be a true negative case, given a negative screen, and thus represents the proportion of true negative cases out all those receiving a negative screen (TN/TN + FN). Table 3, Table 4, Table 5 below provide a visual reference for the diagnostic accuracy calculations using 2x2 confusion matrices.

**Table 3:** 2x2 confusion matrix of ASSIST risk vs. diagnostic interview outcomes at the low-risk threshold for detecting any cases of problematic use with diagnostic accuracy indices.

	No disorder	Any disorder	
No use	True Negative (TN)	False Negative (FN)	NPV (TN/TN + FN)
Any disorder	False Positive (FP)	True Positive (TP)	PPV (TP/TP + FP)
	Specificity (TN/TN + FP)	Sensitivity (TP/TP + FN)

**Table 4:** 2x2 confusion matrix of ASSIST risk vs. diagnostic interview outcomes at the moderate-risk threshold for detecting cases of moderate SUD or harmful use.

	No disorder & Mild SUD/Hazardous use	Mod. SUD/Harmful use & Severe SUD/Dependence	
Low risk	True Negative (TN)	False Negative (FN)	NPV (TN/TN + FN)
Mod. risk	False Positive (FP)	True Positive (TP)	PPV (TP/TP + FP)
	Specificity (TN/TN + FP)	Sensitivity (TP/TP + FN)

**Table 5:** 2x2 confusion matrix of ASSIST risk vs. diagnostic interview outcomes at the high-risk threshold for detecting cases of severe SUD or dependence.

	Non-severe/Non-dependence	Severe SUD/Dependence	
Non-high risk	True Negative (TN)	False Negative (FN)	NPV (TN/TN + FN)
High risk	False Positive (FP)	True Positive (TP)	PPV (TP/TP + FP)
	Specificity (TN/TN + FP)	Sensitivity (TP/TP + FN)	

#### 3.3.3 Likelihood ratios and clinical utility indices

Finally, we will also compute a range of additional metrics, including likelihood Ratios for positive (LR+) and negative (LR-) tests; Clinical Utility Index for positive (CUI+) and negative (CUI-) tests; and Cohen's weighted Kappa (interrater agreement). LR + describes the ratio of true cases to false cases, while LR-describes the likelihood of true non-cases to false non-cases. Higher ratios indicate better accuracy. CUI+ is the product of sensitivity and positive predictive value, while CUI- is the product of specificity and the negative predictive value. Finally, weighted-kappa will be used to indicate the level of agreement between risk outcomes from the ASSIST and diagnostic classifications from the clinical interview.

### 3.4 Cultural acceptability

The final section of the app includes questions to assess cultural acceptability. Participants are asked to rate their agreement to three questions on a 5-point Likert scale, using facial expression emojis, ranging from 0 (frown) to 5 (smile). The three questions are: “How easy was it to use this iPad to answer our questions?“, “Was it okay for us to ask these questions?“, and “Were the questions easy to understand?” Responses will be quantified and used to assess acceptability, together with the time taken to complete the app. In addition, research assistants will be on-hand to ask participants about their qualitative experiences using the app in a semi-structured yarning style discussion.

### **3.5** Sample Size and Power Calculations

For the validity assessments, a series of power calculations were conducted to determine the minimum sample size needed to detect a large between-groups effect, with type-I, and type-II error rates of 5 % (corresponding to α = 0.05), and 10 % (corresponding to an *a priori* power of 90 %) respectively. Where possible, we will also aim to recruit a 1:1 ratio of participants for each group (i.e., positive/negative cases), and so a 1:1 ratio of participants was used here. Using these parameters as default, we conducted the following power calculations in G-power [43].

For each regression model assessing concurrent validity, it was determined that a minimum of 111 participants are needed for each model (corresponding to line-of-best-fit slope of 0.3). For each logistic regression model assessing concurrent validity with the independent assessment, a total sample size of 62 using each substance was determined. The additional parameters used in this analysis included a probability of reaching diagnosis in the diagnosis present group of 0.70 (corresponding to a sensitivity of 70 % and an Odds Ratio of 2.33 [0.70/0.30]), versus a chance probability (i.e., 50%) in the diagnosis absent group. These parameters yielded a total sample size of 62 individuals (approximately 31 in each group diagnosis present/absent assuming 1:1 sampling). For each ANOVA model assessing discriminant validity, a minimum of 96 participants using each substance are required to power the analysis (corresponding to *η*^2^ = 0.14 [44]). Based on this, the minimum number of participants to detect a large effect (Cohens’ *d* > 0.80 [45]) was 38 participants for each group. This corresponds to thirty-eight low risk, thirty-eight moderate risk, and thirty-eight high-risk individuals for each substance (which equals approximately 114 participants for each given substance). For the reliability assessment, we also calculated the minimum sample size required to detect reliability coefficients >0.70, setting the type-I error rate to 0.05, with a minimum power of 0.90, with the number of scale items equal to 4 (tobacco) or 5 (all other substances). This method has been suggested previously [46] and has been used widely in health and medical research settings [47]. The result was a minimum sample size of 38 for a 5-item scale, and 40 for the 4-item scale. We elected to use 40 as the minimum sample size required for consistency.

## 4 Discussion

The Pitjantjatjara ASSIST has been transformed across a number of dimensions to increase its cultural appropriateness. The next step is to assess its validity, reliability and acceptability for use with Indigenous Australian populations. Given the lack of valid, culturally appropriate and reliable instruments for detecting risky substance use, validation of *the app* adds to the growing list of community-driven initiatives aimed at closing the health gap in Australia.

A valid, reliable app detecting risky substance use in Indigenous Australian settings could reduce such use population-wide. In addition, an instrument with a high degree of diagnostic accuracy may be able to provide a greater degree of insight into the prevalence of substance use disorders (including dependence) within the Indigenous Australian population. Assuming *the app* is valid and reliable, future research will also focus on identifying the rate of substance use disorders among the Indigenous Australian community.

While there are a number of strengths of this research, there are also some potential limitations that need to be discussed. Primarily, *the app* relies on accurate self-report data, which can be a challenge in Indigenous Australian settings for a number of reasons, including stigma, shame, cultural sensitivity, and general distrust that occurs around disclosure of substance use [20,21]. *The app* used here however, is designed to address some of these challenges, by prioritising capacity for anonymous, self-assessment and feedback. Additionally, as the app is currently only in Pitjantjatjara and English, it excludes many Indigenous Australians not fluent or literate in these languages. Therefore future research will also be needed to investigate the validity and reliability of both the English version of *the app*, as well as any additional languages that are developed and added in the future.

This project acknowledges the unique challenges and strengths that Indigenous Australian communities face in addressing the harm from substance use disorders. The Pitjantjatjara ASSIST — developed largely by, and with community — serves as a reminder about the role that community should play in the development, implementation and scale-up of culturally-grounded public health strategies.

## CRediT authorship contribution statement

**Matthew W.R. Stevens:** Writing – review & editing, Writing – original draft, Visualization, Validation, Supervision, Software, Resources, Project administration, Methodology, Investigation, Funding acquisition, Formal analysis, Data curation, Conceptualization. **Sue Bertossa:** Writing – review & editing, Project administration, Investigation, Data curation. **Dominic Barry:** Writing – review & editing, Supervision, Data curation. **Chris Holmwood:** Writing – review & editing. **KS Kylie Lee:** Writing – review & editing, Methodology, Investigation, Formal analysis, Conceptualization. **John Marsden:** Writing – review & editing, Validation, Methodology, Investigation, Formal analysis, Conceptualization. **Matt Pedler:** Writing – review & editing, Investigation, Data curation. **Mark Thompson:** Writing – review & editing, Project administration, Data curation, Conceptualization. **Scott Wilson:** Writing – review & editing, Supervision, Project administration, Data curation. **Robert L. Ali:** Writing – review & editing, Writing – original draft, Visualization, Validation, Supervision, Software, Resources, Project administration, Methodology, Investigation, Funding acquisition, Formal analysis, Data curation, Conceptualization.

## Ethics approval and consent to participate

This study has received ethics approvals from the Aboriginal Health Research Ethics Committee (Reference ID: 04-23-1090), Southern Adelaide Clinical Human Research Ethics Committee (Reference ID: 2024/HRE00063) and The University of Adelaide Human Research Ethics Committee (Reference ID: 39,232).

## Consent for publication

Results will be published in peer-reviewed journals, presented at conferences, and shared with participants and community stakeholders via summary reports.

## Trial sponsor

This trial is sponsored by the University of Adelaide. University of Adelaide, Research Office, Adelaide, SA 5005, research@adelaide.edu.au.

## Endnotes

1 Yarning is a culturally sensitive communication method rooted in Indigenous storytelling traditions, increasingly utilised by non-Indigenous Australian researchers and clinicians to facilitate knowledge exchange and build relational spaces with Aboriginal and Torres Strait Islander peoples. It involves a two-way, narrative-based interaction that emphasises listening, questioning, and sharing to elicit more meaningful descriptions of one's experiences, contrasting with Western narrative concepts by prioritising relational dynamics and oral traditions over individualistic exposition.

## Funding

MS, RA and CH are funded by an Australian Government Department of Health and Aged Care Grant (4-HPNM4SN). The funder are not involved in the conduct of this research, including any stages of design, data management, analysis, or publication decisions.

## Declaration of competing interest

The authors declare that they have no known competing financial interests or personal relationships that could have appeared to influence the work reported in this paper.

## Data Availability

The final dataset will be de-identified and made publicly accessible.

## List of Abbreviations

ADAC: Aboriginal Drug and Alcohol Council of South Australia
ASSIST: Alcohol, Smoking and Substance Involvement Screening Test
DASSA: Drug and Alcohol Services South Australia
DSM-5-TR: Diagnostic and Statistical Manual of Mental Disorders (5th Edition, Text Revised)
ICD-11: International Classification of Diseases (11th Revision)
ROC;: Receiver Operating Characteristics curve
SBIRT: Screening, Brief Intervention, Referral to Treatment
SSI: Substance Specific Involvement (score)
SUD: Substance Use Disorder
WHO: World Health Organization
