# Supplementary material for: ASSIST in Pitjantjatjara: Protocol for a randomised crossover validation study among Aboriginal and Torres Strait Islander Australians
Source: Contemp Clin Trials Commun. 2025 Aug 7;47:101532. doi: 10.1016/j.conctc.2025.101532 (PMC12395507; doi:10.1016/j.conctc.2025.101532)
Supplement: Multimedia component 1 [file mmc1.pdf]

Validation of the Pitjantjatjara ASSIST in a Randomized Crossover Clinical Trial: Statistical Analysis Plan

Matthew W. R. Stevens[1], John Marsden[2], Sue Bertossa[3], Dom Barry[4], Chris Holmwood[1], K.S. Kylie Lee[5−9], Matt Pedler[3], Mark Thompson[10], Scott Wilson[11], & Robert L. Ali[1]

[1]School of Biomedicine, The University of Adelaide; [2]NHIR Maudsley Biomedical Research Centre, King's College London; [3]Flinders Wellbeing Centre, South Australia; [4]School of Psychology, University of South Australia; [5−9]Centre for Alcohol Policy Research, La Trobe University; National Drug Research Institute, Curtin University; Faculty of Medicine and Health, The University of Sydney; Burnet Institute, Melbourne; Edith Collins Centre, Sydney Local Health District; [10]Drug and Alcohol Services South Australia; [11]Aboriginal Drug and Alcohol Council South Australia Aboriginal Corporation

July 14, 2025

Contents

1 Introduction . . . **2**

2 Study Design . . . **2**

3 Measures . . . **2**
- 3.1 Pitjantjatjara ASSIST (*index test*) . . . 2
- 3.2 Diagnostic Interview (*reference test*) . . . 3

4 Analysis Objectives . . . **4**

5 Statistical Methods . . . **4**
- 5.1 Concurrent Validity . . . 4
- 5.2 Discriminant Validity . . . 5
- 5.3 Internal Consistency Reliability . . . 6
- 5.4 Test-Retest Reliability . . . 6
- 5.5 Diagnostic Accuracy . . . 7
- 5.6 Cultural Acceptability . . . 7

6 Handling of Missing Data . . . **8**

7 Deviations from Intended Analyses . . . **8**

8 Sample Size Considerations . . . **8**

9 Statistical Software . . . **9**

10 Data Monitoring and Protocol Adherence . . . **9**

11 Amendments . . . **9**

1 Introduction

This Statistical Analysis Plan (SAP) will outline the statistical methods for analyzing data from a randomized crossover clinical validation study of the culturally-adapted Alcohol, Smoking, and Substance Involvement Screening Test (ASSIST) in Pitjantjatjara. The study will involve Pitjantjatjara-speaking Aboriginal and Torres Strait Islander Australians, and will aim to validate the newly developed app as a means for detecting risky substance use for five common drugs: alcohol, tobacco, cannabis, methamphetamine, and inhalants. The SAP is prepared prior to database lock to ensure transparency and reproducibility, which is critical for adherence to ethical approvals from the Aboriginal Health Research Ethics Committee (ID: 04-23-1090), Southern Adelaide Clinical Human Research Ethics Committee (ID: 2024/HRE00063), and The University of Adelaide Human Research Ethics Committee (ID: 39232). The trail protocol was pre-registered on the Open Science Framework (https://doi.org/10.17605/OSF.IO/GNZAY) and Australian New Zealand Clinical Trials Registry (Trial ID: ACTRN12625000413426), and has been peer-reviewed. A pre-print is available via SSRN [1]. All analyses will be conducted using R (version 4.4.3 or higher) with packages including `ggplot2`, `dplyr`, `psych`, `pROC`, and `irr`.

2 Study Design

This is a prospective, randomized, crossover AB / BA clinical trial, conducted in Aboriginal health and welfare settings in remote, rural and urban South Australia. Eligible participants — Pitjantjatjara or English-speaking A<u>n</u>angu men or women, aged 18–65 — will be recruited from sites including Drug and Alcohol Services South Australia (DASSA), Flinders Wellbeing Center, and the Aboriginal Drug and Alcohol Council (ADAC), and DASSA's mobile outreach service in the APY Lands. Participants will complete the Pitjantjatjara ASSIST app (index test) and a yarning-style diagnostic interview with a trained professional (gold-standard reference) in randomized order, with a follow-up app assessment after 7–28 days (ideally 14 days). A subset of high-risk participants in the withdrawal service of DASSA can also undergo an independent clinical evaluation by an Addiction Medicine Specialist, who will be blinded to the results of previous investigations. Data will be collected via REDCap (interview), and on a secure ASSIST database (Pitjantjatjara app) using unique anonymous participant codes.

3 Measures

3.1 Pitjantjatjara ASSIST (*index test*)

The Pitjantjatjara ASSIST is a culturally adapted, digital version of the Alcohol, Smoking, and Substance Involvement Screening Test (ASSIST), translated into Pitjantjatjara to ensure accessibility for A<u>n</u>angu participants [2, 3]. It will assess risky substance use for five substances: alcohol, tobacco, cannabis, methamphetamine, and inhalants. The instrument comprises six items per substance (five for tobacco), each addressing aspects such as frequency of use, cravings, and related harms over the past three months. Items have varying response scales (e.g., 0–2 to 0–8), and responses will be summed to produce a Specific Substance Involvement (SSI) score for each substance. SSI scores range from 0–39 for alcohol, cannabis, methamphetamine, and inhalants, and 0–31 for tobacco, with higher scores indicating greater risk. The instrument is self-administered via a tablet-based app, and incorporates culturally appropriate prompts, language, and visuals developed collaboratively with community input. SSI scores for each substance will be used to inform assessment of validity, reliability, and diagnostic accuracy.

3.2 Diagnostic Interview (*reference test*)

The diagnostic interview using DIS-SAM [4] will serve as the gold standard reference for evaluating the validity of the criterion against the DSM-5-TR and ICD-11 criteria for substance use disorders [5, 6]. The interview will be conducted in a yarning style format to align with cultural communication preferences [7]. The interview will assess substance use disorders (DSM-5-TR) and hazardous use, harmful use, or dependence (ICD-11). Categorical diagnoses will inform discriminant validity and diagnostic accuracy assessments, while symptom/cluster counts will support concurrent validity assessments.

3.2.1 DSM-5-TR Symptoms

For DSM-5-TR, the diagnostic interview will assess 11 symptoms for each substance:

1. Taking the substance in larger amounts or over a longer period than intended.
2. Persistent desire or unsuccessful efforts to cut down or control use.
3. Spending a great deal of time obtaining, using, or recovering from the substance.
4. Craving or a strong desire to use the substance.
5. Recurrent use resulting in failure to fulfill major role obligations at work, school, or home.
6. Continued use despite persistent or recurrent social or interpersonal problems caused or exacerbated by the substance.
7. Giving up or reducing important social, occupational, or recreational activities due to use.
8. Recurrent use in situations where it is physically hazardous.
9. Continued use despite knowledge of a physical or psychological problem likely caused or exacerbated by the substance.
10. Tolerance, defined as needing more of the substance to achieve the same effect or diminished effect with the same amount.
11. Withdrawal, manifested by characteristic symptoms or use of the substance to relieve or avoid withdrawal.

3.2.2 DSM-5-TR Diagnostic Classifications

Diagnoses will be assigned as *no disorder*, *mild substance use disorder* (SUD), *moderate SUD*, or *severe SUD* based on the presence of these symptoms during the interview. Specifically: 0–1 symptom reported indicates no disorder, 2–3 symptoms indicate mild substance use disorder (Mild SUD), 4–5 symptoms indicate moderate SUD, and 6+ symptoms indicate severe SUD).

3.2.3 ICD-11 Symptom Clusters

For ICD-11, symptoms will be grouped into seven independent clusters across three categories:

- **Dependence** (four clusters, corresponding to three symptom groupings): (1) Impaired control over substance use; (2a) Increasing prioritization of substance use over other activities; AND (2b) continued use despite harm to physical, psychological, or social wellbeing; (3) Physiological features (tolerance or withdrawal). ***Note***: For Cluster 2 to be met, both components, a and b, are required.
- **Harmful Use** (one cluster): (1) Has caused physical or mental health consequences to oneself or to others due to their substance use.

- **Hazardous Use** (one cluster): (1) Substance use appreciably increases the risk of harmful physical OR mental health consequences to the user or to others.

3.2.4 ICD-11 Diagnostic Classifications

Diagnoses will be assigned as no disorder, hazardous use, harmful use, or dependence based on the presence of these clusters. To reach a diagnosis of dependence, individuals must endorse two of the three symptom clusters in the past 12 months (Note: cluster 2a and cluster 2b are two constituent parts of a single cluster, and therefore must both be met). For harmful use, individuals must both (1) not meet the criteria for dependence, and (2) meet the symptom cluster for harmful use (i.e., caused harm to self or others). Likewise, to meet the criteria for hazardous use, the individual must both (1) not meet the criteria for either dependence or harmful use, and (2) meet symptom clusters for hazardous use (i.e., increased risk of physical OR mental health consequences to self or others).

Note: Diagnostic categories are essential for clinical research and practice, and therefore this study will primarily focus on validating the ASSIST as a tool for classifying individuals according to previously established symptom-based diagnostic classifications. However, categorical classification can reduce granularity — e.g., someone endorsing six DSM-5-TR symptoms is diagnostically equivalent to someone endorsing all 11 criteria, despite differences in symptom burden. Therefore, to complement the categorical approach, raw symptom counts will also be examined as a more detailed indicator of severity, particularly in the assessment of concurrent validity.

4 Analysis Objectives

1. To confirm the concurrent validity of ASSIST Specific Substance Involvement (SSI) scores with DSM-5-TR and ICD-11 symptom/cluster counts using Spearman's Rho (ρ).
2. To evaluate linear and quadratic relationships between ASSIST SSI scores and DSM-5-TR and ICD-11 symptoms/cluster counts.
3. To confirm the discriminant validity of ASSIST SSI scores across DSM-5-TR and ICD-11 disorder categories.
4. To confirm the internal consistency reliability of ASSIST SSI scores using McDonald's omega (ω).
5. To confirm test-retest reliability of ASSIST SSI scores using intraclass correlation coefficients ($ICC_{(3,1)}$).
6. To confirm the diagnostic accuracy and appropriate cut-off scores of ASSIST SSI scores using Receiver Operating Characteristic (ROC) curve analysis, and related indices.
7. To confirm the cultural acceptability of the Pitjantjatjara ASSIST using Likert scale ratings and qualitative feedback.

5 Statistical Methods

5.1 Concurrent Validity

Objective: To assess associations between ASSIST SSI scores and the number of DSM-5-TR/ICD-11 symptom/cluster counts for each substance (Objective 1).

Confirmatory Analyses: Partial Spearman's rank-order correlations will be calculated between SSI scores (continuous) and DSM-5-TR or ICD-11 symptom/cluster counts (ordinal), controlling for key demographics (age, gender, employment, marital status, and living arrangement) as well as SSI scores for the remaining substances to adjust for poly-substance use. A total of ten partial correlations will be reported (5 substances $\times$ 2 systems). To control for multiple comparisons while maintaining statistical power, the false discovery rate (FDR) will be controlled at 5% using the Benjamini-Hochberg (BH) procedure [8]. The BH procedure is adaptive, and provides a more balanced trade-off between type I and type II error risk than familywise error rate (FWER) controls [9].

Exploratory Analyses: To further explore functional relationships between SSI scores and diagnostic outcomes (Objective 2), linear and quadratic regression models will be fitted to symptom/cluster count data, and logistic regression will be used to model the probability of receiving a high-risk diagnostic status, as determined by an independent medical assessment.

Interpretation: For the confirmatory analyses, correlation strength will be interpreted according to Cohen's guidelines: $\rho \leq 0.20$ (negligible), 0.21–0.39 (weak), 0.40–0.59 (moderate), and ≥ 0.60 (strong) [10]. For the exploratory modelling, model fit will be assessed using R^2 and adjusted R^2 indices, and compared using AIC/BIC indices. For logistic models, odds ratios (ORs) will quantify the change in odds of high-risk diagnostic status per unit increase in SSI scores.

Cut-off: A statistically significant moderate correlation ($\rho \geq 0.50$) will be taken as evidence of concurrent validity, where statistical significance is determined by FDR-controlled results at the 5% level using the BH procedure.

Reporting: For the confirmatory analysis, partial correlation coefficients with 95% confidence intervals and two-tailed BH–adjusted p-values will be reported. For exploratory analyses, regression coefficients (intercepts and slopes), model fit indices (F-statistics, R^2, and adjusted R^2), model comparison metrics (AIC, ΔAIC, BIC, ΔBIC), and, where logistic regression is conducted, odds ratios with 95% confidence intervals and two-tailed p-values will be reported.

5.2 Discriminant Validity

Objective: To evaluate whether ASSIST SSI scores can differentiate between diagnostic classifications under DSM-5-TR and ICD-11 frameworks (Objective 3).

Confirmatory Analyses: One-way ANOVA will assess differences in SSI scores across four diagnostic groups within each substance, for both DSM-5-TR and ICD-11 classifications. Planned pairwise t-tests will compare adjacent diagnostic categories following significant omnibus results. To control the false discovery rate (FDR) arising from multiple comparisons, BH correction will be applied with a fixed threshold of 5%. Where normality assumptions are violated (e.g., for low-frequency substances), appropriate non-parametric tests will be used in place of ANOVA (e.g., Kruskal-Wallis) and pairwise post-hoc tests.

Post Hoc Tests: Planned pairwise comparisons (with BH-correction) will specifically test:

- **DSM-5-TR**: No SUD vs. mild SUD; mild vs. moderate SUD; and moderate vs. severe SUD.
- **ICD-11**: No disorder vs. hazardous use; hazardous use vs. harmful use; and harmful use vs. dependence.

Cut-off: Discriminant validity will be supported when omnibus tests yield statistically significant results (two-tailed p ¡ 0.05), and subsequent planned pairwise comparisons are significant under FDR control at the 5% level via the BH procedure.

Reporting: For each ANOVA, F-statistics (or chi-square [χ^2] statistics for Kruskal-Wallis tests), two-tailed p-values, and eta-squared (η^2) effect sizes with 95% confidence intervals will be reported. For each pairwise t-test, t-statistics, two-tailed BH–adjusted p-values, and Cohen's d effect sizes with 95% confidence intervals will be reported.

5.3 Internal Consistency Reliability

Confirmatory Analyses: McDonald's hierarchical omega (ω_h) will be calculated for each substance as the primary indicator of internal consistency, given the expected multidimensional factor structure [11]. Coefficients will be estimated using nonparametric bootstrapping ($N =$ 10,000 resamples) to obtain empirical distributions and 95% confidence intervals. Hierarchical omega is preferred in this context as it does not assume unidimensionality and offers a more accurate estimate of general factor saturation in multidimensional scales [12, 13].

Supporting Analyses: For completeness, omega total (ω_t) and Cronbach's alpha (α) will also be calculated using the same bootstrap procedure. Although alpha remains a conventional benchmark, it assumes tau-equivalence and may underestimate reliability when this assumption is violated [14].

Interpretation: Reliability coefficients will be interpreted using the following thresholds: $\leq$ 0.49 (poor), 0.50–0.74 (moderate), 0.75–0.89 (good), and $\geq$ 0.90 (excellent). Hierarchical omega will be used as the primary index of scale reliability.

Cut-off: A bootstrapped 95% confidence interval for ω_h that includes or exceeds 0.70 will be taken as evidence of acceptable internal consistency.

Reporting: For each substance, ω_h, ω_t, and α coefficients will be reported with their corresponding bootstrapped 95% confidence intervals and two-tailed p-values.

5.4 Test-Retest Reliability

Objective: To assess stability of ASSIST SSI scores over 7–28 days (ideally 14 days) (Objective 5).

Confirmatory Analyses: Test–retest reliability will be assessed using intraclass correlation coefficients (ICC) for each substance's total SSI score. A two-way mixed-effects model (absolute agreement, single rater/measurement) will be used, appropriate for repeated assessments of the same individuals with the same instrument [15]. Scale-level ICC values will serve as the primary reliability outcome.

Supporting Analyses: Item-level ICCs will also be calculated and reported to provide additional insight into individual item stability over time.

Interpretation: ICC values will be interpreted as follows: $\leq$ 0.49 (poor), 0.50–0.74 (moderate), 0.75–0.89 (good), and $\geq$ 0.90 (excellent) [16].

Cut-off: Test–retest reliability will be considered acceptable if the scale-level ICC is $\geq$ 0.50 and the 95% confidence interval lies entirely within or above the moderate reliability range (i.e., lower bound of CI $\geq$ 0.50).

Reporting: For each substance, ICC coefficients (scale-level and item-level) with 95% confidence intervals and two-tailed p-values will be reported.

5.5 Diagnostic Accuracy

Objective: To assess ASSIST SSI scores' ability to classify/predict DSM-5-TR substance use disorder (SUD), and ICD-11 hazardous use, harmful use, dependence classifications (objective 6).

Risk Threshold Classifications: To increase statistical power, disorder classifications will be aggregated into three binary comparisons:

- *Low-risk threshold*: No disorder vs. any disorder (i.e., mild/moderate/severe SUD; hazardous/harmful use/dependence)
- *Moderate-risk threshold*: No disorder/mild SUD (or hazardous use for ICD-11) vs. moderate/severe SUD (or harmful use/dependence for ICD-11)
- *High-risk threshold*: Non-severe (i.e., no disorder/mild/moderate SUD) vs. severe SUD (or non-dependence vs. dependence for ICD-11)

Confirmatory Analyses: ROC curve analysis will determine optimal cut-off scores for each risk threshold through Youden's Index (J). Based on the optimal cut-off scores, confusion matrices outlining true positives (TP), true negatives (TN), false positives (FP), false negatives (FN) will be calculated. Six matrices will be developed for each substance (3 risk thresholds $\times$ 2 classification systems). Youden's J optimizes the trade-off between sensitivity and specificity, identifying the point on the ROC curve with maximum distance from the diagonal. Area under the curve (AUC) values and comprehensive diagnostic accuracy indices will assess clinical utility [17].

Supporting Analyses: Individual disorder classifications (no disorder, mild, moderate, severe SUD; no disorder, hazardous use, harmful use, dependence) will also be analyzed and reported to provide granular diagnostic information. Cohen's Kappa (κ) will also be calculated and reported based on the updated cut-off scores.

Interpretation: Statistically significant (two-tailed p ¡ 0.05) AUC $\geq$ 0.71 will indicate better-than-acceptable diagnostic accuracy. Cohen's $\kappa \geq 0.60$ will be used to indicate substantial agreement.

Diagnostic Indices:

- Confusion matrix (TP, TN, FP, FN)
- Sensitivity (Sn: TP/(TP+FN)), Specificity (Sp: TN/(TN+FP))
- Positive Predictive Value (PPV: TP/(TP+FP)), Negative Predictive Value (NPV: TN/(TN+FN))
- Positive/Negative Likelihood Ratios (LR+: Sn/(1-Sp), LR-: (1-Sn)/Sp)
- Diagnostic Odds Ratio (DOR: (TP/FN)/(FP/TN))
- Clinical Utility Index (CUI+: Sn $\times$ PPV, CUI-: Sp $\times$ NPV)
- Cohen's kappa (κ)

Reporting: AUC values with 95% confidence intervals and associated two-tailed p-values will be reported for each risk threshold and disorder-level analysis, alongside optimal cut-offs and complete diagnostic indices including DORs for all comparisons.

5.6 Cultural Acceptability

Objective: To assess cultural acceptability of the ASSIST app (Objective 7).

Method: Mean and SD of 5-point Likert scale ratings (1 = frown, 5 = smile) for three questions on technological ease of use, appropriateness, and understanding will be calculated. Shapiro-Wilk will test normality; if non-normal, medians and IQR will be reported instead. Completion time (mean/SD or median/IQR) will be summarized. Any qualitative feedback will be thematically analyzed.

Justification: Mixed-methods approach will provide both quantitative metrics and qualitative insights into cultural acceptability, essential for indigenous health tools.

Cut-off: Mean ratings above 3.0 — i.e., positive acceptability scores — will indicate appropriate cultural acceptability.

Reporting: Mean (or median) ratings, SD (or IQR), 95% CIs, completion times, qualitative themes (if any).

6 Handling of Missing Data

Missing data will be evaluated using Little's Missing Completely At Random (MCAR) test [18]. If missingness is ¡5%, complete case analysis will be used. For 5–20% missingness, multiple imputation with 5 imputations using predictive mean matching will be applied. Sensitivity analyses will compare complete case and imputed results. A case-by-case decision for exclusion or mean imputation will be considered based on missing data patterns.

Note: The instrument and interview have been designed with forced responses to reduce likelihood of missing data. However, it is possible that some participants may withdraw during data collection, or may be unable to complete one or more components of the study. In cases where missing data occurs within an assessment, i.e., partial completion of the ASSIST or interview — data from that participant will be excluded. However, for participants that complete multiple assessments, but fail to complete the follow-up for example, imputation will occur as described above.

7 Deviations from Intended Analyses

Prior to conducting each assessment, checks for normality and any other assumptions will be conducted. However, in the event that any of the planned assessments indicate substantial deviation from required analytic assumptions (e.g. zero-excess, or non-linearity), appropriate modifications to the analysis strategy will be implemented and fully described in the final analysis report.

8 Sample Size Considerations

Power calculations were conducted using G*Power [19] with $\alpha = 0.05$ and 90% power (i.e., $1 - \beta$), targeting a 1:1 ratio of positive/negative cases where applicable. Stratified sampling will be used to target specific areas where substance use is more prevalent.

- **Concurrent Validity**: To detect a partial correlation of $\rho \geq 0.5$ with multiple control variables, 36 participants per substance will be required, totaling 180 participants (36 $\times$ 5).
- **Discriminant Validity**: To detect a large effect size (η^2 ¿ 0.14) in ANOVA, 96 participants per substance are needed. For pairwise comparisons with Holm correction, 38 participants per risk group per substance (d ¿ 0.8), totaling 114 per substance or 570 participants will be required.

- **Internal Consistency Reliability**: To detect ω ¿ 0.7 with 5–6 items, 40 participants per substance, totaling 200 participants will be needed.
- **Test-Retest Reliability**: To detect ICC $\geq$ 0.5, 40 participants per substance, totaling 200 participants will be required.
- **Diagnostic Accuracy**: To detect AUC $\geq$ 0.71, 44 participants per substance per risk threshold, totaling 660 participants (i.e., 44 $\times$ 3 $\times$ 5) will be needed.

A target of 660 participants is set to meet the largest requirement (diagnostic accuracy), with recruitment continuing until 44 participants per substance/risk combination (15 combinations) are achieved, or until deemed unnecessary by investigators.

Note: Given the likelihood that some participants will have used more than one substance (and therefore will fall into more than one category), reaching the adequate number of participants in each substance/risk combination is likely to occur well before the total sample reaches 660.

9 Statistical Software

Analyses will be conducted in R (version 4.4.3 or newer) with `psych` (alpha, omega), `pROC` (ROC), `lme4` (ICC), and `mice` (imputation). Data will be managed in REDCap.

10 Data Monitoring and Protocol Adherence

A data monitoring team (MS, RA) will oversee trial conduct, safety, and protocol adherence. Monthly audits and check-ins with research assistants will ensure compliance. Protocol deviations will be reported to ethics committees within 72 hours.

11 Amendments

Any amendments to this SAP will be documented, justified, and approved by the Chief Investigators (RA, JM) and Coordinating Principal Investigator (MS) prior to database lock.

References

[1] Stevens, M. W. R., Bertossa, S., Barry, D., Holmwood, C., Lee K.S, K., Marsden, J., Pedler, M., Thompson, M., Wilson, S., & Ali, R. (2025). Assist in Pitjantjatjara: An Order-Randomised Validation Study Protocol [research protocol]. Available at SSRN: https://doi.org/10.2139/ssrn.5262422.

[2] WHO ASSIST Working Group. (2002). The Alcohol, Smoking and Substance Involvement Screening Test (ASSIST): Development, reliability and feasibility. *Addiction, 97*(9), 1183–1194. https://doi.org/10.1046/j.1360-0443.2002.00185.x.

[3] Stevens, M. W. R., Barry, D., Bertossa, S., Thompson, M., & Ali, R. (2022). First-stage development of the Pitjantjatjara translation of the World Health Organization's Alcohol, Smoking and Substance Involvement Screening Test (ASSIST). *Journal of the Australian Indigenous HealthInfoNet, 3*(4), 2. https://doi.org/10.14221/aihjournal.v3n4.2.

[4] Compton, W. M., Cottler, L. B., Hilsenroth, M., & Segal, D. L. (2004). The diagnostic interview schedule (DIS). *Comprehensive Handbook of Psychological Assessment, 2*, 153–162.

[5] American Psychiatric Association. (2022). *Diagnostic and Statistical Manual of Mental Disorders* (5th ed., text rev.). American Psychiatric Publishing.

[6] World Health Organization. (2019). *International Statistical Classification of Diseases and Related Health Problems* (11th ed.). World Health Organization.

[7] Teasdale, K. E., Conigrave, K. M., Kiel, K. A., Freeburn, B., Long, G., & Becker, K. (2008). Improving services for prevention and treatment of substance misuse for Aboriginal communities in a Sydney Area Health Service. *Drug and Alcohol Review, 27*(2), 152–159. https://doi.org/10.1080/09595230701829447.

[8] Benjamini, Y., & Hochberg, Y. (1995). Controlling the false discovery rate: a practical and powerful approach to multiple testing. *Journal of the Royal statistical society: series B (Methodological), 57*(1), 289–300. https://doi.org/10.1111/j.2517-6161.1995.tb02031.x.

[9] Verhoeven, K. J., Simonsen, K. L., & McIntyre, L. M. (2005). Implementing false discovery rate control: increasing your power. *Oikos, 108*(3), 643–647. https://doi.org/10.1111/j.0030-1299.2005.13727.x.

[10] Guilford, J. P. (1950). *Fundamental Statistics in Psychology and Education* (2nd ed.). McGraw-Hill.

[11] McDonald, R. P. (2013). *Test theory: A unified treatment.* psychology press.

[12] Revelle, W., & Zinbarg, R. E. (2009). Coefficients alpha, beta, omega, and the glb: Comments on Sijtsma. *Psychometrika, 74*(1), 145-154. https://doi.org/10.1007/s11336-008-9102-z.

[13] Zinbarg, R. E., Revelle, W., Yovel, I., & Li, W. (2005). Cronbach's α, Revelle's β, and McDonald's ω_h: Their relations with each other and two alternative conceptualizations of reliability. *psychometrika, 70*(1), 123–133. https://doi.org/10.1007/s11336-003-0974-7.

[14] Streiner, D. L. (2003). Starting at the beginning: An introduction to coefficient alpha and internal consistency. *Journal of Personality Assessment, 80*(1), 99–103. https://doi.org/10.1207/S15327752JPA8001_18.

[15] Weir, J. P. (2005). Quantifying test-retest reliability using the intraclass correlation coefficient and the SEM. *Journal of Strength and Conditioning Research, 19*(1), 231–240. https://doi.org/10.1519/00124278-200502000-00038.

[16] Koo, T. K., & Li, M. Y. (2016). A guideline of selecting and reporting intraclass correlation coefficients for reliability research. *Journal of Chiropractic Medicine, 15*(2), 155–163. https://doi.org/10.1016/j.jcm.2016.02.012.

[17] Hanley, J. A., & McNeil, B. J. (1982). The meaning and use of the area under a receiver operating characteristic (ROC) curve. *Radiology, 143*(1), 29–36. https://doi.org/10.1148/radiology.143.1.7063747.

[18] Little, R. J. A. (1988). A test of missing completely at random for multivariate data with missing values. *Journal of the American Statistical Association, 83*(404), 1198–1202. https://doi.org/10.1080/01621459.1988.10478722.

[19] Faul, F., Erdfelder, E., Lang, A.-G., & Buchner, A. (2007). G*Power 3: A flexible statistical power analysis program for the social, behavioral, and biomedical sciences. *Behavior Research Methods, 39*(2), 175–191. https://doi.org/10.3758/BF03193146.
