# Supplementary material for: ASSIST in Pitjantjatjara: Protocol for a randomised crossover validation study among Aboriginal and Torres Strait Islander Australians
Source: Contemp Clin Trials Commun. 2025 Aug 7;47:101532. doi: 10.1016/j.conctc.2025.101532 (PMC12395507; doi:10.1016/j.conctc.2025.101532)
Supplement: Multimedia component 2 [file mmc2.docx]

# Supplementary File 1

## The Pitjantjatjara ASSIST

### Design and development

The Pitjantjatjara ASSIST (“*the app*”) has undergone extensive co-design and consultation with community to ensure its cultural acceptability [1]. For the purposes of this research (i.e., validating the instrument), *the app* was designed for use on an iOS tablet (iPad Air). However, *the app* is also available on Android tablet devices. The decision to develop *the app* exclusively for tablet, rather than for smartphone, was recommended by community leaders to ensure that the font-sizing was appropriate to aid literacy issues and those with visual impairments.

The back-end functionality of *the app* was designed so that, when connected to the internet, data can be collected from each participant before uploading it to a secure, cloud-based storage system located at the University of Adelaide. Due to the potential for internet access issues in rural and remote communities, *the app* stores data locally (i.e., on the device’s internal memory), and uploads the data when internet access is restored.

### App flow

The structure of *the app* includes two components; the ASSIST questionnaire, and tailored feedback on risk. However for the purposes of the research project, a number of additional modifications have been made, including a consent page, demographic questionnaire, and a small exit survey. When users first open *the app*, they are prompted to answer a series of four questions to create a unique code (see *Unique participant code* subsection below). Once all details have been entered, a ‘Participant Information’ button becomes accessible, which then steps individuals through a consenting process. Participation in the study can only proceed once the individual has listened to, or read the study information sheet, and agreed to participate. Participants must agree by checking an on-screen checkbox and clicking the ‘I agree’ button to continue (see Figure S1).

Once consent has occurred, participants proceed to the demographics section of *the app*. The first question asks the individual to identify their gender, which for the purposes of this study, is either male or female^[[1]](#footnote-1)^. Once an individual has indicated their gender, *the app* then guides the individual through a series of demographic questions, related to their living arrangements, primary language spoken at home, current employment status, and relationship status. *The app* then asks questions related to the individual’s substance use (the Pitjantjatjara ASSIST), before providing individuals with tailored feedback about their scores, and associated level of risk (see examples in Figure S2). The gender which participants indicate in the initial stage is used to determine the gender of the voiceover, and graphics which participants are exposed to throughout the remainder of *the app*.


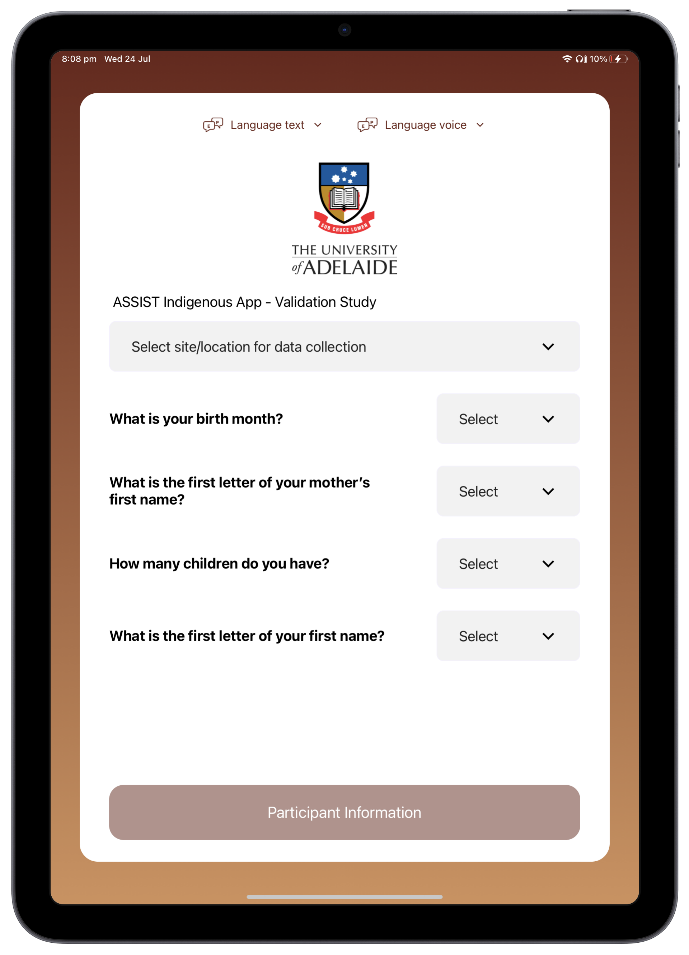

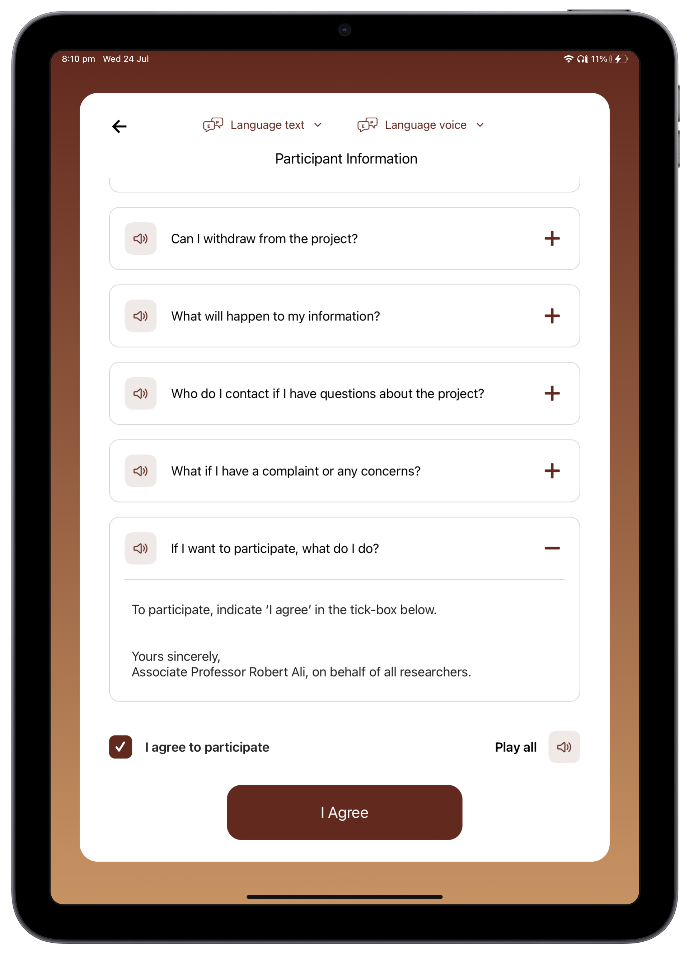


**Figure S1.** Unique participant code creation stage (Left-hand panel); and Participant Information and Consent page (Right-hand panel).


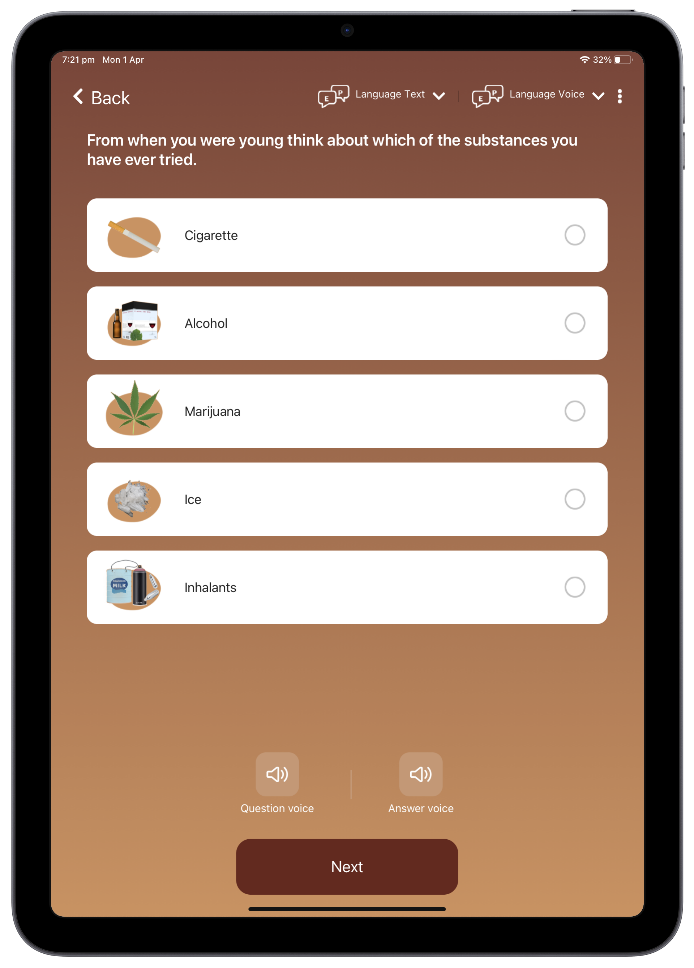

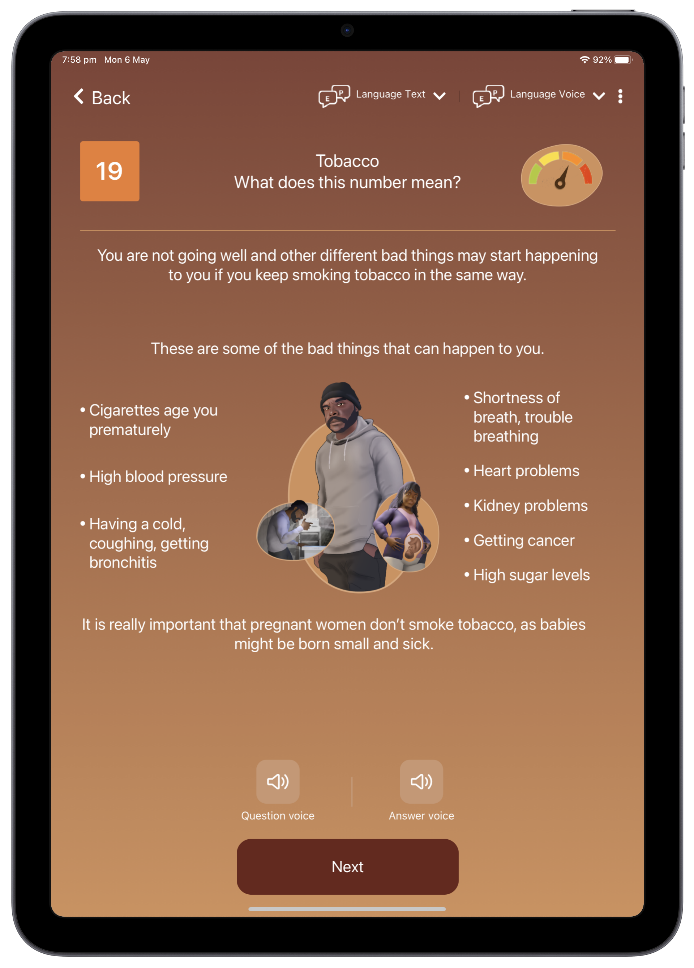


**Figure S2.** Initial substance identification question (Q1) of Pitjantjatjara ASSIST (Left-hand panel); and feedback for a male, moderate risk tobacco user (Right-hand panel).

### App design features

*The app* was co-designed with community to ensure features to improve accessibility, functionality and interpretation of the questions. For example, the addition of graphics depicting each of the substances in common modes; various scenarios depicting the questionnaire items; and some of the potential harms encountered as a result of use. All graphics were developed by a prominent Aboriginal Australian artist.

*The app* also includes an audio voiceover, so the user can listen and read the questions simultaneously. Community experts also recommended that the ability to disaggregate the visual and audio components should also be available, so that users could listen in English and read in Pitjantjatjara, or vice versa. The gender of the voice which participants hear spoken to them throughout *the app*, as well as the characters represented in the graphics they see, are matched to the participant’s gender. Currently, *the app* is limited to English and Pitjantjatjara languages. However, the integration of additional languages spoken by Indigenous Australian communities is planned for the future.

Once the participant has completed the ASSIST part of the questionnaire, they then receive tailored feedback about their scores, level of risk associated, and advice on reducing risk. Future work will focus on integrating a brief intervention (yet to be developed). The brief intervention component will include a series of pre-recorded videos of an Aboriginal health worker talking down the lens of the camera (e.g., to the user) about their level of use and ways to cut-down or stop. The brief intervention will follow the FRAMES model (Feedback, Responsibility, Advice, Menu of options, Empathy and Self-efficacy) [2], and will be adapted using the Indigenous Australian Social and Emotional Wellbeing Model [3], and the *Brief Yarns on Alcohol* video series [4]. Information provided during the ASSIST questionnaire will be used to tailor which video the individual sees as part of the brief intervention. The brief intervention component is currently under development, and will be assessed for acceptability and effectiveness in a later study.

# References

1. Stevens MW, Barry D, Bertossa S, Thompson M, Ali R. First-Stage Development of the Pitjantjatjara Translation of the World Health Organization’s Alcohol, Smoking and Substance Involvement Screening Test (ASSIST). Journal of the Australian Indigenous HealthInfoNet. 2022;3(4):2.
2. Bien TH, Miller WR, Tonigan JS. Brief interventions for alcohol problems: a review. Addiction. 1993 Mar;88(3):315-36.
3. Dudgeon P, Bray A, D'costa B, Walker R. Decolonising psychology: Validating social and emotional wellbeing. Australian Psychologist. 2017 Aug 1;52(4):316-25.
4. Conigrave K, Assan R, Perry J, Bruning G, Hayman N, Wilson S, Lee K. Brief yarns on alcohol. Brief yarns on alcohol. 2023 Jul 31.

1. An awareness and understanding of the need for gender diversity and inclusivity is important. However, during the course of cultural adaptation of the instrument, consultation with opinion leaders and experts from the Pitjantjatjara community identified that inclusion of additional genders beyond male and female would lead to confusion among participants (See Stevens, et al., 2022). [↑](#footnote-ref-1)
